# Tracing the Evolution of the Angiosperm Genome from the Cytogenetic Point of View

**DOI:** 10.3390/plants11060784

**Published:** 2022-03-16

**Authors:** Natalia Borowska-Zuchowska, Magdalena Senderowicz, Dana Trunova, Bozena Kolano

**Affiliations:** Plant Cytogenetics and Molecular Biology Group, Institute of Biology, Biotechnology and Environmental Protection, Faculty of Natural Sciences, University of Silesia in Katowice, Jagiellonska 28, 40-032 Katowice, Poland; magdalena.senderowicz@us.edu.pl (M.S.); dana.trunova@gmail.com (D.T.)

**Keywords:** cytogenetics, genome evolution, genome size, genome downsizing, karyotype evolution, chromosome number, dysploidy, polyploidy, FISH, GISH

## Abstract

Cytogenetics constitutes a branch of genetics that is focused on the cellular components, especially chromosomes, in relation to heredity and genome structure, function and evolution. The use of modern cytogenetic approaches and the latest microscopes with image acquisition and processing systems enables the simultaneous two- or three-dimensional, multicolour visualisation of both single-copy and highly-repetitive sequences in the plant genome. The data that is gathered using the cytogenetic methods in the phylogenetic background enable tracing the evolution of the plant genome that involve changes in: (i) genome sizes; (ii) chromosome numbers and morphology; (iii) the content of repetitive sequences and (iv) ploidy level. Modern cytogenetic approaches such as FISH using chromosome- and genome-specific probes have been widely used in studies of the evolution of diploids and the consequences of polyploidy. Nowadays, modern cytogenetics complements analyses in other fields of cell biology and constitutes the linkage between genetics, molecular biology and genomics.

## 1. Introduction

The plant genome has been analysed at various levels from the native DNA sequence through the chromatin up to the highly condensed metaphase chromosomes. Cytogenetics enables the plant genome to be analysed at all of the aforementioned levels using broad range of approaches and methods. In 1842, the Swiss botanist Karl Nägeli first discovered chromosomes in pollen [1]. Three decades later, the German anatomist Walter Flemming observed chromosomes during cell division for the first time [2]. The early cytogenetic studies used simple chromosome staining methods that stain the chromosomes uniformly, e.g., basophilic aniline dye, acetoorcein/acetocarmine and the Feulgen reaction [3,4]. Chromosome counts constituted the focal point of cytogenetic studies until 1968 when the modern chromosome banding methods were introduced [5]. These technics opened new perspectives in the comparative analyses of plant karyotypes that included tracking any chromosomal changes. However, the development of DNA:DNA *in situ* hybridisation (ISH) brought cytogenetics studies to the next level and tightly linked them with molecular biology [6]. ISH enabled the physical mapping of specific DNA sequences on both chromosomes and interphase nuclei, and indeed, the position of the DNA sequence of choice can be traced throughout the entire cell cycle [7]. Hence, comparative cytogenetics has obtained new, advanced tools for plant evolutionary studies [8,9,10,11]. Nowadays, deciphering the mechanisms that are behind the different evolutionary scenarios require the use of multiple approaches from different disciplines. Modern molecular cytogenetics is among the main players in such studies. Despite the current age of high-throughput Next Generation Sequencing (NGS) technologies, cytogenetics, which enables studies *in situ*, still provides valuable information on the genome structure and evolution. Here, we review the possibilities of using molecular cytogenetic studies on various aspects of the evolution of the angiosperm genome, including karyotype structure, genome size and polyploidy.

## 2. Genome Size Analyses

Genome size refers to the DNA content in the nucleus and is known to be associated with the nucleus and cell size, division rate, and hence, to various organism-level traits such as metabolism, body size or developmental rate [12]. The 1C-value is an amount of DNA in the haploid unreplicated nucleus (holoploid genome size) [13]. It is typically measured in picograms (pg) for mass or as a total number of nucleotides in the megabase pairs (Mbp) where 1 pg is equal to 978 Mbp of DNA [14]. Genome size has mainly been estimated using two cytogenetic methods: flow cytometry and the Feulgen microdensitometry [15,16]. Several technical factors have an impact on generating a reliable genome size estimation, such as selecting the appropriate standard species, nuclei isolation and staining methods or presence of secondary metabolites in the tissues to be analysed (for more details and recommendations for the best-practice approaches, please refer to Doležel et al. [16]). In addition to the technical issues, other factors such as the occurrence of B chromosomes or the presence of individuals at different ploidy levels in one species can also affect the flow cytometry results [17,18]. While intraspecific polymorphisms in ploidy levels are relatively easy to detect using flow cytometry, the occurrence of B chromosomes increases the estimated genome size of only several percent (e.g., 8.7% in *Crepis capillaris*, 6.6% in maize and 5.4% in rye; [19,20,21]) and must be verified by chromosome counting. The rapid advances in DNA-sequencing technology, which have been accompanied by the development of bioinformatic tools, also enables the estimation of genome size directly from the whole-genome data [22,23].

### 2.1. The Patterns and Directions of Genome Size Evolution

There is an astonishing variation in genome size in land plants, which ranges from 0.065 pg in *Genlisea tuberosa* to 152.23 pg in *Paris japonica* [24,25]; however, the median genome size for angiosperms has been estimated to be ca. 1.7–2.5 pg (in the C-value database, the data on genome sizes represent only ~3.1% of plant species) and the majority of species are characterised by having small to very small genomes (1C DNA < 3.5 pg; [26,27,28]). The species with large or very large genomes (1C DNA > 14 pg) are in the minority and they are mostly restricted to a few evolutionary lineages [26,27]. Monocotyledons are relatively abundant in species with large genomes. This clade consists of many species with very large genomes, e.g., *Fritillaria*, *Paris* and *Trillium*, which have 1C DNA > 35 pg [29,30]. Among eudicots, the largest genomes that have been measured to date have been those of the *Viscum* species from the family Santalaceae [27,31]. Recent analyses have suggested that high rates of genome size evolution promoted high rates of diversification and speciation. Within angiosperms, the highest rate of genome size evolution was found within monocots, particularly Poaceae, which also exhibits the highest rate of speciation [32].

The results of genome size measurements when analysed in the phylogenetic background enable the trends and patterns of the genome size changes that accompanied the evolution of different taxa to be hypothesised. Different plant families have different patterns of genome size distribution and evolution [28]. In the family Brassicaceae, a nearly 30-fold variation in genome size was revealed [33]; however, most species fall into the small or very small genome category (less than 3.5 pg/1C according to the classification of Leitch et al. [26]), except for two polyploids *Crambe cordifolia* and *Hesperis matronalis*, which had a medium genome size. The median 1C-value for Brassicaceae is 0.72 pg [33]. A decrease in genome size accompanied the evolution of approximately 50% of the species in Brassicaceae taxa that have been analysed. The other species had an increase in genome size; however, this was quite moderate with significant increases in C-value restricted to only two tribes, Anchonieae and Physarieae [34]. An even larger, 139-fold variation in genome size, was reported for the family Asteraceae [33]. The values of the holoploid nuclear DNA amount in this family varied and ranged from 1C DNA = 0.22 pg in the diploid *Erigeron canadense* to 32.75 pg in the decaploid *Crepis barbigera* [35,36,37]. The analyses showed that genome size does not evolve evenly across the phylogeny of Asteraceae and both increases and decreases in genome size were observed and the directions of evolution had clade-specific patterns [36]. To date, the highest (230-fold) variation in genome size was found in the monocot family Melanthiaceae, which includes *Paris japonica*, the plant species with the largest genome size. However, the median holoploid genome size (5.4 pg) for this family falls into the medium-size category [33]. The main trend of genome size in Melanthiaceae was towards a decrease in genome size and there was only one evolutionary lineage with a striking DNA accumulation in Parideae (the clade that comprised, among others, the genus *Paris*, which has very large genomes) [30].

A relatively high variation in genome size was also observed among closely related species that belong to one genome, e.g., in the genus *Genlisea*, in which except for *G. tuberosa*, which has the smallest genome size (1C DNA = 0.065 pg), its other species had a significantly larger genome size 1C DNA = 1.76 pg (*G. lobate*), which makes about a 27-fold difference between the smallest and the biggest genome in *Genlisea* [24]. However, there are genera that have very small variations in their genome size, e.g., all *Fritillaria* species have very large genome sizes with nearly a three-fold variation [38]. Similar relatively low variations (1C DNA between 0.267–0.705 pg) of genome sizes were reported for the Dipterocarpaceae family of pantropical trees, which have a very small genome size in which a decrease in genome size is the general trend [39].

### 2.2. The Repetitive Sequences—The Main Players in Genome Size Evolution

Several mechanisms have been proposed as contributing to the large variation in genome size among angiosperms. The increases in genome size seem to mainly be the results of the amplification of repetitive sequences and polyploidisation. Polyploidisation had occurred frequently throughout the evolution of angiosperms but it was usually followed by rapid genome reorganisation, which often led to genome downsizing and there is no significant positive linear correlation between genome size and polyploidsation (for more details please refer to Section 4) [40,41]. The major impact on genome size variation in plants as well as in all eukaryotes seems to have been the amplification/elimination of repetitive sequences, especially mobile elements [41]. The number of repetitive sequences differs significantly among species and the average content of repetitive sequences reach from approximately 14% to around 80–90% in plants. The majority (more than 75%) of this repetitive content was identified as being mobile elements [42,43]. Although the mechanisms that are responsible for the decreases in genome size are less recognised, they have been shown to involve recombination-based processes, for example, an unequal recombination or an illegitimate recombination (reviewed by Grover and Wendel [28,44]).

The advances in NGS technologies with their bioinformatic tools that use a graph-based sequence clustering algorithm (RepeatExplorer) have facilitated *de novo* repeat identification and enabled a comprehensive characterisation of repetitive DNAs with information on both the types of repetitive sequences and on their relative proportions in many different plant genera [45]. Several reports have been published that compared the abundance and composition of the repeatomes among a group of closely related species, which showed a relatively high variability in genome size. In the *Hesperis* clade (Brassicaceae) and the tribe Fabeae (Fabaceae), the increases in genome size were mainly caused by the proliferation of the Ty*3-gypsy* elements, particularly an increase of the elements from the Ogre/Tat lineage [9,46], while the retrotransposons of a Chromovirus lineage were proliferated in *Solanum*, *Helianthus* and *Passiflora* species with relatively largest genome size [47,48,49]. Fewer reports have shown increases in genome size as a result of the amplification of the Ty1-*copia* elements [50]. All of the aforementioned plant genera consist mostly of species with small and medium-sized genomes (up to ca. 10 Gbps). The comparative analyses showed that the diversity of mobile elements was comparable among most of the studied species from one genus, however, their abundances differed significantly among the species. Therefore, the genomes had a relatively small number of specific repeats occupying a large proportion of a repeatome [9,43,46,47,48,50,51]. Repetitive sequences in species with genome sizes in this range are reported to be rapidly turning over with half-lives of tens of thousands to a few million years and the recent amplification of retrotransposons are mainly responsible for the genome size while the ancient retrotransposons usually account for a small proportion of the genome [43,52,53].

In species with a genome that is larger than 10 Gbp such as *Fritillaria* or Norway spruce, a repeatome comprises many different repeat families and a highly heterogeneous, relatively low-abundance degraded repetitive DNA [38,43,54]. These degraded repeats result from the accumulation of point mutations, indels and rearrangements. The changes may be so severe that they turn repeats into unique or low-copy sequences [43]. No predominant repeats that tracked the increasing/decreasing trends of the evolution of genome size were revealed in *Fritillaria* [29,38], which supports a scenario in which amplified repeats constantly accumulate owing to the rare removal of DNA. An absence of the elimination and low turnover of repetitive DNA are the main contributors to the evolution of extremely large genomes and show that their size cannot simply be explained by the activity of a few high-abundance repeat families [43,54].

The proliferation of tandem repeats has a much smaller impact on the variation in genome size when compared with retrotransposons [43]. The highly amplified satellite families contributed significantly to the increase in genome size in *Heloniopsis umbellata* [55], *Fritillaria affinis* [29] and *Oenothera biennis* [56].

The molecular cytogenetic method, i.e., the fluorescence *in situ* hybridisation, enables not only the visualisation of the particular repetitive sequences in the genome but also allows to determine their chromosomal distribution. In species characterised by a small genome size, the repetitive sequences are mostly localised in the pericentromeric heterochromatin [57]. In species with large genome size, however, the patterns of heterochromatic bands on the chromosomes can be more diverse, e.g., in *Fritillaria* species [38]. Moreover, FISH enables the comparative studies of the repetitive sequences chromosomal distribution in the related taxa. Orzechowska et al. [58] showed that even if the particular repeat is present in the genomes of several related species, its chromosomal organisation can differ even in karyotypes of closely related species.

## 3. Why Is the Chromosome Number So Variable in Angiosperms?

The chromosome number is the primary feature in plant cytogenetics, however, chromosome number is still known for only ca. 20–30% of angiosperms [59]. The current coverage differs among the various taxonomical groups. Within the 20 largest angiosperm families, the best-studied family is Apiaceae for which counts have been obtained for 42% of the species in the taxa, while the least-studied family is Bromeliaceae for which the chromosome number has only been established for 7% of the species. The coverage for the largest plant family, Asteraceae, is 32% of the species [59]. The data that has been obtained thus far indicate a huge variation in the number of chromosomes that ranges from *n* = 2, e.g., *Brachycome dichromosomatica* [60] to *n* = ca. 320 for *Sedum suaveolens* [61]. The evolution of the chromosome number in plants was mainly driven by two mechanisms: polyploidy (the duplication or multiplication of whole chromosome sets) and dysploidy (the change in the basic chromosome number that can occur either in the sense of an increase (ascending dysploidy) or a decrease of the basic chromosome number (descending dysploidy) resulting from Robertsonian fusion–fission rearrangements but also from intra- or interchromosomal translocations, deletions) [62,63]. Aneuploidy (the gain or loss of one or more chromosomes) has a lower impact on chromosome variability [64], since monosomics and nullisomics are often lethal in diploid lineages. Moreover, plants with an additional one or more chromosomes usually suffer from a fitness disadvantage due to an imbalanced gene content and have diminished fertility due to the irregular chromosome segregation [65]. There are a few described species whose evolution seems to involve aneuploid chromosome number changes, e.g., *Amaranthus caudatus*, which includes two cytotypes, 2*n* = 32 and 2*n* = 34, for which the second cytotype is suggested to be tetrasomic (2*n* + 2) [66,67].

### 3.1. Changes in the Chromosome Number against the Phylogenetic Background

Although plant chromosome count reports are still published nowadays, more often the data on chromosome numbers are combined with the results of other methods, for example, molecular cytogenetic and phylogenetic ones or molecular biology [18,68]. An analysis of the chromosome number combined with molecular phylogenetic data enables the accurate interpretation of the cytological information in a phylogenetic context and reveals the various patterns of the evolution of the chromosome number in different evolutionary lineages. The ancestral haploid chromosome number *n* = 7 for angiosperms has been suggested based on previous comparative cytogenetic surveys [69,70]. Recently, these results were supported by Carta et al. [71], who modelled the evolution of the haploid chromosome number in angiosperms based on the data that is available in the Chromosome Count Data Base [59]. Similar values of the ancestral state of the chromosome number have also been inferred for several families in the angiosperms, for example, the basic chromosome number *x* = 10 was revealed for Eleusininae [72], *x* = 9 for Melanthiaceae, Asteraceae [30,73,74] and *x* = 7 for Brassicaceae [34]. Some other families have an ancestral chromosome number that seems to be the duplicate of a number of the angiosperm ancestral state such as Araceae (*n* = 16 or *n* = 18 [75] or Arecaceae (*n* = 16; [76]). Analyses of the evolution of the chromosome number that is based on the datasets for all of the angiosperms or analyses on the family level have implied that dysploidy is the most important mechanism in the evolution of the chromosome number in both the deep and shallow nodes while polyploidysation events have mainly been inferred on the tips of the tree branch [71]. Conversely, a phylogenetic approach that was integrated with a comparative genomic study in plants revealed recurrent whole genome duplication (WGD) events throughout the evolution of plants [77,78]. An ancient genome duplication predated angiosperm diversification and further polyploidy events were shared by major lineages of flowering plants [79]. Two polyploidy events in monocots have been inferred to have pre-dated the diversification of Poaceae as well as one triplication event that is probably shared by all core eudicots [78]. Further WGDs are also shared by several major clades of eudicots, including Asteraceae [80], Brassicales [81] and Legumes [82]. It is impossible to detect these ancient polyploidisation events using only cytogenetic methods because of the genomic and chromosome restructuring that follow polyploidisation [83].

**Figure 1:**
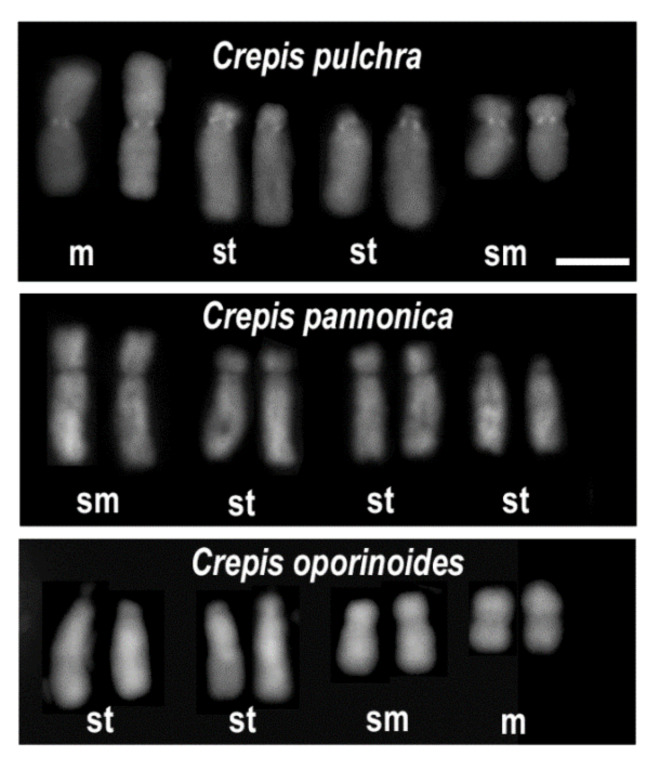
Karyograms of *Crepis* species with 2*n* = 2*x* = 8 chromosomes from different evolutionary lineages based on [18]. Although they have the same chromosome number, each species has a different karyotype formula. Letters below each pair of chromosomes indicate the type of chromosome: m—metacentric; sm—submetacentric and st—subtelocentric. Scale bar = 5 µm.

Most families of angiosperms that have been described include species that have several different basic chromosome numbers as well as different ploidy levels [74,84,85]. Published data showed that there are no general patterns in the changes in the chromosome number although descending dysploidy and polyploidy seems to be most frequent [30,74,86]. In some genera such as *Chenopodium* (*x* = 9) or *Solanum* (*x* = 12), the basic chromosome number is the same or nearly the same for all of the species but many polyploidisation events have been inferred [87,88,89,90]. Other genera such as *Crepis* include species with a different basic chromosome number (*x* = 3, 4, 5, 6 and 11) while there are relatively few polyploids [18,91]. There are also many genera where both mechanisms seem to be responsible for the variation in chromosome number in a similar way [92,93]. Ascending dysploidy seems to have been a more rare event in the evolution of angiosperms than descending dysploidy. Exemplary, in the Marantaceae family, there were ten events of decreasing chromosome numbers and only one event of ascending dysploidy [94]. However, in the family Arecaceae or in the genus *Passiflora*, ascending dysploidy appears to be the predominant direction of chromosomal change [51,76]. In the evolution of taxa with more than one basic chromosome number, each number can occur several times, e.g., in genus *Crepis*, the basic chromosome *x* = 4 evolved at least ten times and the differences in the structure of the karyotype among the species from different evolutionary lineage are easy to notice (Figure 1; [18])

### 3.2. Genome Evolution from the Cytogenetic Point of View

Chromosome evolution has been studied most comprehensively in two families that encompass cultivated and model species: Poaceae (e.g., *Brachypodium distachyon* and *Oryza sativa*) and Brassicaceae (*Arabidopsis thaliana*) [92,95,96]. Multicolour FISH with BAC (bacterial artificial chromosomes) clones has enabled comparative analyses of *Brachypodium* chromosomes that have revealed that descending dysploidy, a common trend in this genus, primarily occurs via nested chromosome fusions [92,97]. Although several basic chromosome numbers have been retrieved for *Brachypodium*, all of them are lower than the *x* = 12 of the Intermediate Ancestral Grass Karyotype (IAGK) [98]. The ancestral *Brachypodium* karyotype seems to have *x* = 10 and two events of nested chromosome fusion have been suggested to have accompanied its evolution [92]. Such a chromosome set is present only in the tetraploid *B. mexicanum*. Next, centric fusion gave rise to karyotypes with *x* = 9, which is present in several perennial diploid species (e.g., *B. sylvaticum* and *B. pinnatum*). Then, an end to end fusion of two chromosomes resulted in the karyotype with *x* = 8, which is present in *B. glaucovirens* [92]. In the evolution of the karyotype of *Arabidopsis thaliana* and related species, other mechanisms, e.g., a pericentric inversion that generated acrocentric chromosomes and a subsequent reciprocal translocation between two chromosomes (one or both acrocentric) was shown to be most common [11,84]. The dysploidy has been reported for many other genera, such as *Hypochaeris* [99] and *Reichardia* [100]. In the case of the latter, it was shown that the rDNA sequences, the constitutive heterochromatin and the GC-rich DNA are implicated in chromosomal rearrangements during the dysploidy events in *Reichardia* [100].

Recently, other approaches such as FISH using painting oligo probes has accelerated research on the chromosome rearrangements that accompanied the evolution of several different genera [101]. An oligo pool that is designed from a single-copy DNA sequence can be used as hybridisation probes that are specific to a chromosomal region (or regions) [87], to a whole chromosome arm [102] or to an entire chromosome [103]. Recently, oligo–FISH barcoding pools were developed for several mainly cultivated species, which enabled the detection of various chromosomal rearrangements that accompanied their evolution and speciation [87,104,105]. However, there are some taxa such as *Citrus* and *Populus* that seem to have very conserved karyograms. Comparative analyses using oligo painting probes that were specific to each chromosome did not reveal any interchromosomal translocation [103,106].

**Figure 2:**
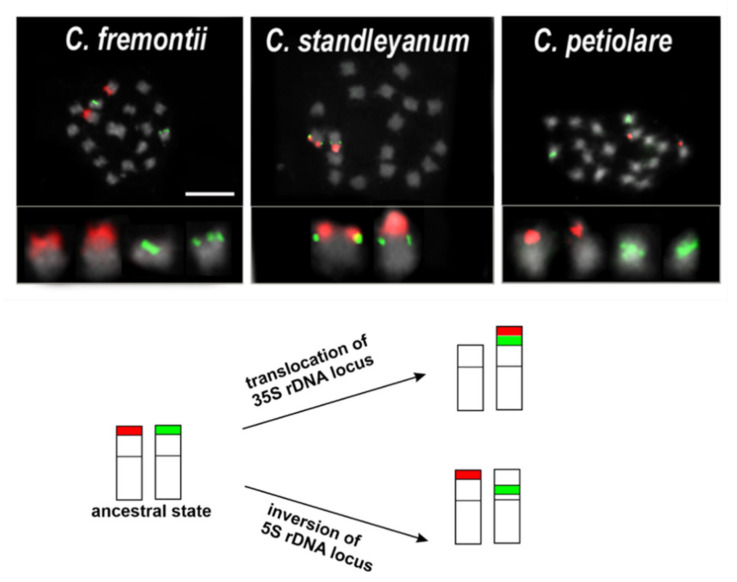
Karyotypes representing each variant of the rDNA loci distribution in the diploid *Chenopodium* species with genome A based on [89,108]. The ancestral state that was inferred for this clade was one locus of each 35S (red fluorescence) and 5S rDNA (green fluorescence) locus placed in two different chromosomes in a subterminal position like in *C. fremontii*. The loci pattern observed in *C. standleyanum* might be explained by the translocation of the 35S rDNA locus to the chromosome with a 5S rDNA array located in a subterminal position, whereas a plausible explanation for the rDNA patterns in *C. petiolare* (5S rDNA is in a more proximal position) seems to be an inversion of the part of the chromosome arm with subterminally located 5S rDNA. Scale bar = 5 µm.

The oligo-based chromosome painting technique should be applicable to any plant species with a sequenced genome and it is likely that the oligo probes that are designed from one species would be useful in other related species [101]. In the wild and non-model genera for which the data on genome sequences are not available, the chromosomal markers (barcodes) such as various repetitive sequences (rDNA, satellite repeats and telomeric repeats) have been used in comparative analyses. There were many studies that have used the repetitive sequences as FISH probes, which enabled the chromosome identification and the comparative analyses of the chromosome structure among related species [107]. The most often used chromosomal markers are rDNA sequences, which enable the chromosome rearrangements in many groups of closely related species to be hypothesised (Figure 2; [89,108,109,110]). Although the rDNA loci have been shown to be excellent chromosome markers, many species have intraspecific polymorphisms in both their number and localisation, and therefore, different accessions of the same species could have different patterns of rDNA loci [66,111,112].

Telomeric sequences are predominantly present in the chromosome termini; however, several species with interstitially located telomeric sequences have been described [107]. In some species, such a locus could be a trace of the chromosome end to end fusion, translocation or inversion [62,107,113]. However, in others the interstitial telomeric sequences could be inserted via a translocation with mobile elements or by a mechanism of the rolling-circle replication of extrachromosomal circular DNA [62,107,114]. Regardless of its origin, when the interstitial locus of telomeric repeats exists, it is often a very good chromosome marker [104]. The rDNA sequences and telomeric repeats are evolutionary conserved and, once they are isolated and cloned, can be used to analyse a wide variety of plant species while satellite repeats are specific to a species or to a small groups of closely related taxa [107,115].

Recently, the development of NGS techniques has enabled tandem repetitive sequences from different wild and cultivated plant genera to be isolated and characterised [116]. Combining the phylogenetic reconstructions of the relationships of species with the cytogenetic approaches is a powerful way to decipher the evolutionary events that are associated with genome divergence [117]. Based on such analyses, the structural karyotype changes and the pathways for chromosome dysploidy have been hypothesised for many groups of plant species [89,118,119].

## 4. Polyploidy in Angiosperms

### 4.1. Introduction to Polyploidy: ‘Definitions’ and ‘Numbers’

**Figure 3:**
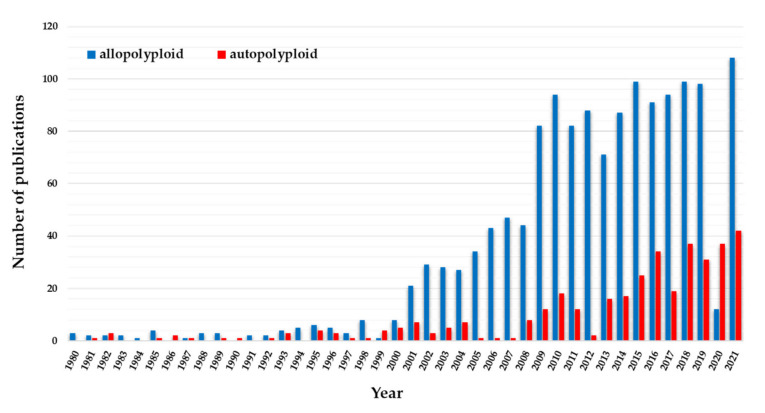
The number of publications on auto- and allopolyploids between 1980 and 2021 based on PubMed (https://pubmed.ncbi.nlm.nih.gov/about/; accessed on 18 January 2022, National Center for Biotechnology Information (NCBI) at the U.S. National Library of Medicine).

Polyploidy, a process in which three or more haploid chromosome sets are present within a single nucleus, has repeatedly influenced the evolution of all angiosperms [120,121,122]. The initial estimations of the number of polyploids among flowering plants, which have mainly been based on chromosome counts, genome size assessments and stomatal guard cell measurements ranged from ~30% to ~70% [121,123,124]. The first classification of polyploids, which was proposed by Kihara and Ono almost a century ago [125], distinguished two classes: (i) the autopolyploids that derive from the chromosome doubling of a single individual and (ii) the allopolyploids that derive from hybridisation. According to a more recent definition, autopolyploid formation is accompanied by a genome doubling that occurs within one species; however, it may involve either a single individual or a cross between two individuals of the same species that represent genetically distinct lineages (e.g., species with the genome composition AA doubles to become the autotetraploid AAAA) [126,127]. In contrast, allopolyploid formation involves an interspecific hybridisation followed by genome doubling (e.g., AA × BB → AB → AABB) [121,122,126,128]. Both neopolyploids and relatively recent polyploids can be classified into either of the aforementioned classes. Historically, allopolyploids were considered to be more common than autopolyploids [124]. Moreover, many economically important plants such as bread wheat, tobacco, oilseed rape, cotton, banana and coffee have an allopolyploid origin. Thus, allopolyploids have received much more attention in the scientific community than autopolyploids (Figure 3), which for many years were considered to be an evolutionary dead-end that could rarely lead to further diversification [127]. Although the scientific interest in the evolutionary studies on autopolyploids has increased significantly in the last two decades, research on allopolyploids/hybrids is still outpacing research on the autopolyploids (Figure 3).

The introduction of genomic approaches has shed more light on the impact of polyploidy in angiosperm evolution by revealing the WGDs in the ancestry of plants with the earliest one (known as the ζ event) that occurred before the divergence of gymnosperms and angiosperms [78,120,129]. The discovery that all flowering plants have experienced polyploidy in their evolutionary history made classifying plants as ‘diploids’ or ‘polyploids’ more complicated. For instance, it was shown that there were at least three WGD events in the evolutionary history of the dicot model plant, *Arabidopsis thaliana*, two recent WGDs (α and β) within the Brassicaceae lineage and the triplication event (γ) that is shared by all core eudicots [77,78]. Thus, the ‘pure’ diploid nature of *A. thaliana* that is based on its chromosome number, genome size and gene copy number has been revisited. *A. thaliana*, like many other paleopolyploid species (or ancient polyploids), underwent a diploidisation process that involved a dramatic reorganisation at the genome, chromosome and gene levels in order to restore a diploid-like behaviour [122,130]. Such a post-polyploid genome divergence is often associated with descending dysploidy, which changes the chromosome number back to a diploid-like one [131,132]. For example, chromosome painting revealed that the karyotype of the Australian crucifers (*Stenopetalum nutans*, *Stenopetalum lineare* and *Ballantinia antipoda* that have *n* = 4, 5 and 6, respectively) descended from an ancestor with *n* = 8 through allopolyploid WGD, which was followed by a large-scale decrease in their chromosome number [10]. Based on their work on wheat, Feldman and Leavy [133] classified the alterations that the newly formed allopolyploid had to face into two groups: (i) the revolutionary changes that occur immediately after polyploidisation and involve chromosomal translocations, elimination of low-copy DNA sequences, amplification/reduction/elimination of high-copy sequences, gene elimination and alterations in epigenetic patterns and gene expression, and (ii) the evolutionary changes that occur during a longer evolutionary timeframe and may involve translocations between the subgenomes; introgressions from different, closely related polyploids or diploids that result in the recombinant genomes appearance; gene inactivation and the functional diversification of homoeoalleles through mutations. Many outstanding comprehensive reviews focus on the different aspects of polyploid evolution and their significance [121,122,126,128,129,133,134]. The current review summarises the use of cytogenetic tools in studies of polyploidy in plants from the ‘classical’ chromosome observations to the modern FISH modifications that enable ‘tracking’ their evolutionary history.

### 4.2. Cytogenetic Approaches on Duty in Polyploid Research

#### 4.2.1. ‘Hunting’ for Polyploids—Cytogenetic Methods in Polyploid Identification

The cytogenetic methods have ‘accompanied’ the polyploid studies from the very beginning. Traditionally, polyploids were primarily identified based on their morphological parameters (e.g., larger flowers, leaves with an altered length-to-width ratio, heavier fruits, altered size and density of the stomata [124,135,136]). Since the use of the morphological characters as polyploidy markers may be misleading [137], additional analyses have to be conducted in order to directly assess the ploidy level, e.g., chromosome counting and/or measuring the nuclear DNA content [124,138,139]. However, the accurate determination of the ploidy status of the studied individual usually requires the use of multiple approaches. For instance, simultaneously determining the DNA content together with chromosome counting and stomatal cell measurements enabled the discovery of a recent autopolyploidy in a representative of Orchidaceae, *Vanilla planifolia* [140]. Even though the chromosome counts and genome size estimations using flow cytometry are still considered to be good approaches in identifying polyploids, they do not always provide a direct answer about the type of polyploidy. For example, a trio of annual *Brachypodium* species (Poaceae) with 2*n* = 10, 20 and 30, respectively, were initially described by Robertson [141] as an autopolyploid series of *B. distachyon* with a base chromosome number *x* = 5 based on the simple chromosome counts. The use of modern cytomolecular methods, i.e., genomic *in situ* hybridisation (GISH) and FISH with BAC-based probes provided evidence that these cytotypes have to be considered as distinct species [57,142]. The cytotypes with 2*n* = 10 and 2*n* = 20, which were initially described as a diploid and an autotetraploid, respectively, are two diploid species with *x* = 5 (*B. distachyon*) and *x* = 10 (*B. stacei*). The use of the total genomic DNAs of both identified diploids as probes in *in situ* hybridisation clearly identified these two species as the putative ancestors of the allotetraploid *B. hybridum* with *x* = 5 + 10 (a cytotype that was initially described as autohexaploid) [57]. These data, supported by a phenotypic characterisation, flow cytometry assessment of genome sizes, and molecular phylogenetic analyses, enabled the three aforementioned species to be distinguished [143].

**Figure 4:**
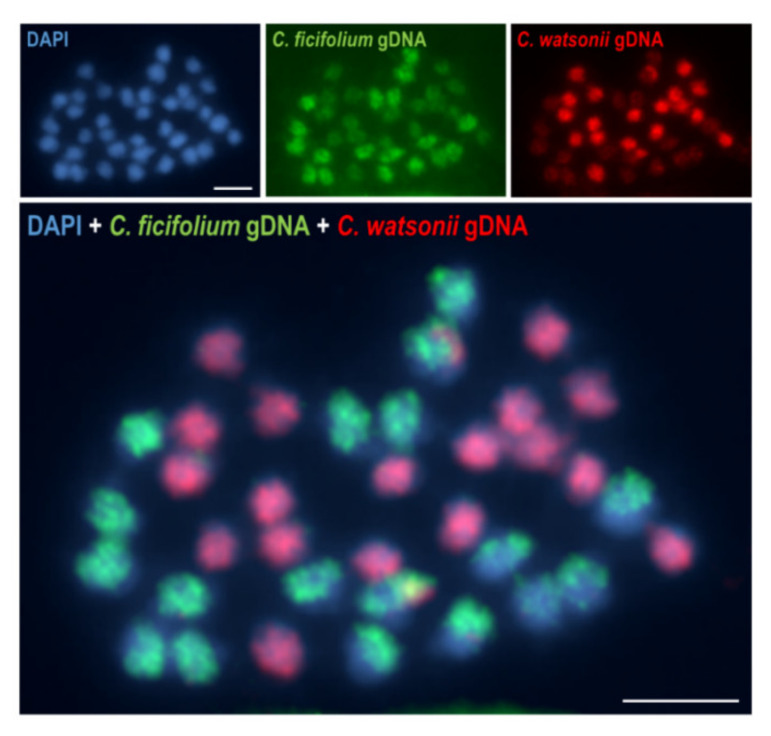
Mitotic metaphase chromosomes of the allotetraploid *Chenopodium berlandieri* (AABB) after double GISH with gDNA that had been isolated from the diploid *C. ficifolium* (BB; green fluorescence) and the diploid *C. watsonii* (AA; red fluorescence) based on [108]. Scale bars 5 µm.

GISH was first used in a study of the subgenomic organisation of *Hordeum* × *Secale* intergeneric hybrid in 1989 [144]. Since then, this method has been widely used to determine the ancestral/parental genomes in the hybrids and allopolyploids [145] that belong to genera such as *Arabidopsis* [146], *Brassica* [147,148], *Coffea* [149], *Gossypium* [150], *Nicotiana* [151], *Tragopogon* [152], *Spartina* [153] and many others. For example, using GISH, a putative diploid parental species has been proposed for the allotetraploid *Chenopodium berlandieri* (genome composition AABB): a B genome donor similar to *C. ficifolium* and an A genome donor similar to *C. watsonii* (Figure 4) [108].

The successful differentiation of the subgenomes on the chromosomes and interphase nuclei of the polyploids using GISH largely depends on the presence of the genome-specific repetitive sequences [154]. When there are very close affinities among the diploid progenitors, the distinguishing between the donor subgenomes using GISH can be challenging. Such a simultaneous discrimination of the three different subgenomes of the allohexaploid *Triticum aestivum* L. (bread wheat; 2*n* = 6*x* = 42; genome composition AABBDD) turned out to be difficult [155]. Additional modifications of the GISH protocol that involved the preannealing of labelled DNA probes and the prehybridisation of the chromosomal samples with the blocking DNA, which were introduced by Amosova et al. [156], enabled the reproducible discrimination between closely related subgenomes. An alternative approach for ‘painting’ the subgenomes in bread wheat was successfully applied by Zhang et al. [157], who used BAC-FISH with three BAC clones that contained dispersed repeats that preferentially hybridise to either the A- or the D-genome.

#### 4.2.2. Polyploidisation Events and What Happens Next?

An analysis of more than 10,000 genome sizes of angiosperms (based on data from the Plant DNA C-values database [23]) revealed the tendency towards genome downsizing, i.e., the majority of the analysed species had genomes that were smaller than expected when considering the polyploidisation events in their evolutionary history [158]. For example, this trend was observed in most of the polyploid species of *Avena* [159], *Brassica* [160], *Triticum* and *Aegilops* [161,162] and many others. It has been proposed that the DNA loss in allopolyploids resulted in a divergence of the homoeologous chromosomes, and therefore it restores the diploid-like behaviour during meiosis [163,164]. However, a few polyploids that have been studied represent the opposite trend, i.e., an increase in the amount of DNA relative to the respective diploids, e.g., the *Nicotiana* polyploids that were formed ~1–5 Mya [165]. In newly formed polyploid species (or the relatively recent ones), however, the additivity in the DNA amount relative to their ancestors can frequently be observed [40]. For instance, no significant loss of DNA following autopolyploidy was observed in *Vanilla planifolia* in which the DNA content increased proportionally with the ploidy level: from ~2.52 pg/1C in the diploids through ~3.84 pg/1C in the autotriploids and up to ~5 pg/1C in the autotetraploid accessions [140]. Much attention has been paid to discovering the mechanisms that might induce genome restructuration, thus leading to genome downsizing (general trend) or increasing (rare scenario). Additionally, the question of what kinds of sequences undergo elimination/amplification in a polyploid nucleus is of particular interest. To address these questions, the cytogenetic and genomic approaches were employed in studies on polyploids of different evolutionary ages starting from the resynthesised forms and neopolyploids through the relatively recent polyploids to the ancient ones. 

There are several examples of neopolyploids that were formed within the last 150 years, e.g., *Cardamine schulzii* [166], *Senecio cambrensis* and *S. eboracensis* [167], *Spartina anglica* [168], *Tragopogon mirus* and *T. miscellus* [169]. These polyploid systems represent extraordinary evolutionary models mainly because they were formed recently, their parental species are known, and, in many cases, they are characterised by multiple origins. These features of the neopolyploids permit: (i) a better understanding of allopolyploid formation from the very beginning and (ii) the verification of whether the evolution is repetitive or not. Two allotetraploid *Tragopogon* species were formed in the Palouse region (eastern Washington and western Idaho, USA) within the last 90 years: *T. mirus* (2*n* = 4*x* = 24), which was derived from a cross between *T. dubius* × *T. porrifolius* and *T. miscellus* (2*n* = 4*x* = 24), which originated from *T. dubius* × *T. pratensis* [170]. The application of GISH enabled the ancestral subgenomes in both allotetraploids to be determined, however, an extensive chromosomal polymorphism between different individuals was revealed, including intergenomic translocations and aneuploidy, which were manifested by the presence of monosomic and trisomic plants [171]. Aneuploidy was found to be frequent in all of the populations of *T. miscellus* [172] and *T. mirus* [152] that have been studied to date. Interestingly, the plants that exhibited aneuploidy were either characterised by the expected chromosome number through reciprocal monosomy-trisomy/nullisomy-tetrasomy of the homoeologues (compensated aneuploidy) or the loss of homoeologues (non-compensated aneuploidy). Thus, the chromosomal variation, which was still present in the approx. 40-generation-old *Tragopogon* plants, revealed that the genome instability after WGD can be significant and prolonged [172]. In contrast to *Tragopogon* and many of the resynthesised allopolyploids that exhibit rapid structural changes of the merged genomes [171,173,174,175,176], there are examples of polyploids that show relative genome stability, e.g., the allododecaploid grass, *Spartina anglica*, that was formed in the 19th century [153,177] and the evolutionarily older grass, the allotetraploid *Brachypodium hybridum* ([8,97] with the exception of rDNA sequences) which was formed ~1.4 and ~0.14 Mya. Also, the allotetraploid cotton (*Gossypium*; evolutionary age ~1.5 Myr) for many years has been considered as relatively ‘quiescent’ concerning rapid genome rearrangements, as was shown by AFLP genomic loci analysis [178] and GISH [150]. However, the subgenome stability in cultivated cotton has been revisited in recent years. The use of NGS technologies (e.g., single-molecule long-read (PacBio) and Hi-C sequencing) in the assembly of the genomes of the cotton cultivars revealed a number of genetic variations, including inversions, translocations and the intergenomic transfer of centromeric retroelement from genome D to A [179,180,181]. Unlike for *Gossypium*, solely cytogenetic approaches were very efficient in detecting genome rearrangements in many polyploid systems. For example, comparative C-banding and FISH with its modifications (primarily GISH) enabled numerous chromosomal translocations in polyploid wheat species and their relatives to be identified [182,183,184,185,186]. 

Among the proposed mechanisms that can lead to duplications, deletions and translocations in allopolyploids are the homoeologous exchanges that are caused by a meiotic mispairing between homoeologues (chromosomes originating from different subgenomes but showing a substantial similarity) for review see [126]. These homoeologous exchanges have been observed in both synthetic polyploids [173,176,187,188] and recent polyploids [172,189] due to their meiotic instability. These ‘meiotic irregularities’ such as the formation of uni- and multivalents, laggard chromosomes and chromosome bridges were first observed in polyploids using simple cytogenetic chromosome stainings. Polyploids were often classified as either auto- or allopolyploids based on the frequency of multivalents during diakinesis and metaphase I [190]. It was proposed that autopolyploids often suffer from the formation of multivalents during meiosis. In contrast, allopolyploids were considered to be more stable because of their diploid-like pairing behaviour, at least when the ancestral species were distantly related. In many cases, however, the genome divergence between progenitors was insufficient to avoid the pairing of homoeologues and therefore new mechanisms were required to stabilise the homologous pairing [191]. The established polyploids, including allohexaploid *Triticum aestivum* and autotetraploid *Arabidopsis arenosa*, behave like diploids during meiosis (i.e., the presence of bivalents only at metaphase I). It was shown that the major chromosome pairing locus, *Ph*1, which is located on the long arm of chromosome 5B, facilitates the prevention of non-homologous pairing between three subgenomes of bread wheat [192,193,194]. In the autopolyploids such as *A. arenosa*, however, it was proposed that the increase of crossing-over interference supports the formation of bivalents over multivalents [195,196]. Unlike the established polyploids, the resynthesised forms [171,176,197] and neopolyploids [171,172] suffer from the numerous ‘meiotic irregularities’ that accompanied their early generations. The use of GISH and FISH with the chromosome- and genome-specific BACs as probes enable the identification of particular chromosomes at different stages of meiosis, and therefore, enable the determination of homoeologous chromosome pairs in different allopolyploids. The recent studies of Xiong et al. [198] revealed a higher number of meiotic errors in the resynthetic forms of *Brassica napus* (genome composition AACC) than in their natural counterparts. The most commonly observed irregularities at diakinesis involved non-homologous centromere associations, a homoeologous recombination that resulted in translocations and associations of the 35S rDNA loci followed by their breakage. Interestingly, it was also revealed that chromosomal inheritance is strongly correlated with the level of synteny between the homoeologous chromosomes, i.e., the homoeologues that share synteny along their entire length (e.g., chromosomes A1/C1; A2/C2 [184]) formed multivalents more frequently and had a polysomic inheritance at telophase I [198]. The frequency of chromosome mispairings, however, might depend on the particular resynthetic line, e.g., the frequency of A-C bivalents in resynthetic *B. napus* varied from ~4% [198] up to ~47% [176] of the analysed pollen mother cells. 

Studies on many resynthesised and natural allopolyploids have shown the frequent elimination of homoeologous sequences, including the single-copy protein-coding genes [161,199,200] and repetitive sequences, e.g., retrotransposons, satellite repeats [58,175] and the tandemly-repeated 35S and 5S ribosomal RNA genes (rRNA genes) [108,201,202,203,204,205]. The rDNA coding sequences (18S, 5.8S, 25S and 5S rDNA) are highly conserved even between phylogenetically distant taxa, and therefore they are frequently used as probes in FISH. In contrast, the non-coding rDNA sequences, the internal transcribed spacers (ITS1 and ITS2) and the intergenic spacer (IGS), are significantly more diverse (for review see [115,206,207]). Initially, rDNAs were considered to be the markers of polyploidy. The hybrid origin of many plant species has been documented using the ITS sequences [208]. However, an increasing number of reports on different polyploids have revealed either uniparental losses of rDNA loci [108,202,209,210] or intergenomic homogenisation via concerted evolution [211], which limits the use of sole rDNAs as polyploidy indicators. There are at least three possible evolutionary scenarios for the 35S rDNA loci in the allopolyploids [207]: (i) the inheritance of all of the ancestral rDNA loci without any significant changes in their structure [212]; (ii) the uniparental inheritance of the rDNA loci (as an effect of rDNA loci conversion or elimination from one subgenome) [88,210,211,213,214] or (iii) the uniparental inheritance of the rDNA loci followed by structural changes that result in the formation of the new rDNA classes [215]. The genus *Nicotiana* constitutes an excellent model in studies of the fate of ancestral rDNA in allopolyploids at different evolutionary ages, including the young allotetraploids that were formed <200,000 years ago: *N. tabacum*, *N. rustica* and *N. arentsii* and ‘old’ allopolyploids from the sections *Polydicliae* (estimated to have formed ~1 Mya) and *Repandae* (evolutionary age ~4–5 Myr) [216]. As has been revealed using FISH, all young *Nicotiana* allotetraploids had the sum of 35S and 5S rDNA loci that had been expected from the numbers that were observed in their progenitors [213,217]. In the ‘old’ *Nicotiana* allopolyploids, however, the diploidisation process was accompanied by the appearance of unique rDNA families, which differed from their putative diploid progenitors and a 35S and 5S rDNA loci loss, i.e., a reduction of rDNA loci to a ‘diploid-like’ number [215]. Further, molecular analyses that used Southern blot hybridisation revealed an intergenomic homogenisation of 35S rDNA that was either complete (*N. arentsii*) or partial (*N. rustica* and *N. tabacum*) [213,216]. Taking into account other polyploid systems such as *Gossypium* [211], *Brassica* [205], *Thinopyrum* [218] and *Atropa* [214], it can be concluded that the 35S rDNA copies from one ancestral species were frequently overwritten or even entirely substituted by the rDNA variants that were derived from the second ancestor in the process of sequence conversion. Even though there are examples of the immediate 35S rDNA rearrangements that start after the formation of a hybrid [219,220], the intergenomic homogenisation of 35S rDNA seems to be a time-dependent process. Although the precise mechanisms that lead to this homogenisation via concerted evolution are poorly understood, gene conversion and unequal crossing-over are thought to play a major role in this process [221,222]. Unlike 35S rDNA, the homogenisation of 5S rDNA seems to occur within a single array with no (or negligible) exchange between the loci [218,223,224]. 

**Figure 5:**
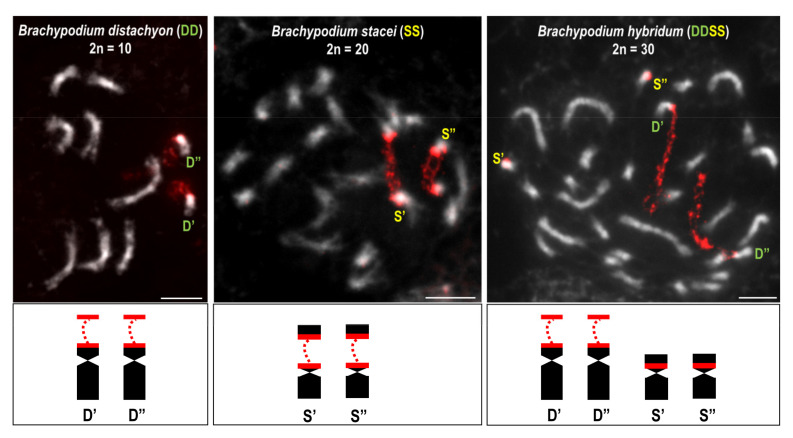
FISH with 25S rDNA (red fluorescence) as a probe on the mitotic prometaphase chromosomes of two diploid and one allotetraploid *Brachypodium* species (*B. distachyon* and *B. stacei* are considered to be the putative progenitors of the allotetraploid *B. hybridum* based on [57,97,111,201]. Note the presence of secondary constrictions on one chromosomal pair in each species. The highly condensed rDNA loci in *B. hybridum* (derived from *B. stacei* and marked as S’ and S”) are transcriptionally silenced via nucleolar dominance, thus they do not form secondary constrictions. D’ and D”—the *B. distachyon* or *B. distachyon*-inherited 35S rDNA loci; S’ and S”—the *B. stacei* or *B. stacei*-inherited 35S rDNA loci. Scale bars 5 µm.

Polyploidisation is also accompanied by an epigenetic repatterning. It has been demonstrated that changes in both the DNA methylation patterns as well as the histone modifications in polyploids are linked with: (i) the (re)activation of the transposable elements that might occur immediately after WGD and (ii) changes in the gene expression patterns [225]. The latter have been observed in numerous plant taxa, including *Arabidopsis* [226], *Brassica* [173,227,228], *Tragopogon* [229], *Gossypium* [230], *Triticum* [231,232] and many others. Moreover, it was revealed that the reactivation of the transposable elements, e.g., the long terminal repeats retroelements (LTRs), can affect the expression patterns of neighbouring genes in polyploids [233,234]. At least one prominent change in the homoeologue expression pattern in an allopolyploid organism can be observed at the cytological level. This phenomenon, which has been termed nucleolar dominance (also known as ‘differential amphiplasty’), was first observed by Navashin [235] and describes the selective silencing of the 35S rRNA gene loci that had been derived from one progenitor in a hybrid [236,237]. The lack of secondary constrictions on the chromosomes in which the under-dominant rDNA loci are located constitutes a cytological manifestation of their transcriptional repression, e.g., in several genotypes of the allotetraploid *Brachypodium hybridum*, only the *B. distachyon*-inherited 35S rDNA loci were able to form secondary constrictions, and therefore were transcriptionally active. In contrast, the repressed *B. stacei*-derived loci remained condensed (Figure 5) [111,201,238]. Among the classical cytogenetic methods, silver staining enables the actively transcribed 35S rRNA genes to be visualised in situ [239]. The simultaneous use of silver staining and FISH with the rDNA coding sequences as probes (exclusively 18S or 25S rDNA) has been used to determine the ancestral rDNA expression in the root-tip cells of several *B. hybridum* genotypes. The uniparental expression of the *B. distachyon*-inherited 35S rDNA loci in *B. hybridum* was manifested by the presence of the Ag-NOR bands [238]. As has been shown in *Brassica* [205,240,241], *Arabidopsis* [242] and *Brachypodium* [243], nucleolar dominance is a tissue-specific, fully reversible phenomenon, and therefore the under-dominant rRNA genes could be reactivated at certain developmental stages. Much attention has been paid to uncover the molecular mechanisms that underlies nucleolar dominance. Studies on both dicot and monocot allopolyploids have revealed that the epigenetic changes play a crucial role in the inactivation of the rRNA genes via nucleolar dominance [201,244,245,246,247,248,249,250,251]. For instance, in the allotetraploid *Arabidopsis suecica*, the inactive 35S rDNA loci that had been derived from *A. thaliana* were associated with a high level of DNA methylation, especially within the promoter regions, and a heterochromatic histone modification, the dimethylation of lysine 9 of histone H3 (H3K9me2) [248]. Similarly, a higher methylation level at the DNA motifs, CHG and CHH and the presence of the heterochromatic histone marks, H3K9me2 and H3K27me3, were observed for the A-genome 35S rDNA loci in a resynthetic allotetraploid wheat, which led to their transcriptional silencing and further elimination that started with the S4 generation [252]. Moreover, it has also been shown that the repression of the 35S rRNA gene loci was accompanied by an RNA-dependent DNA methylation pathway (RdDM) in *A. suecica* [253,254]. The precise mechanisms that determine which ancestral loci will undergo silencing still remain elusive. It has been documented that nucleolar dominance in allopolyploids is not a maternal effect [240] and also seems to be independent of the rDNA copy number [255]. In some systems such as *Arabidopsis* and *Hordeum*, the 35S rDNA loci are transcriptionally repressed based on their chromosomal position [256,257], thus the impact of the neighbouring DNA sequences on the rRNA gene expression should be considered.

## 5. Conclusions

The molecular cytogenetic approaches significantly contributed to the current understanding of the plant genome structure, function and evolution. As next-generation sequencing costs have continued to decrease, which makes NGS more affordable and accessible, the number of species with sequenced genomes has dramatically increased [258]. The availability of the whole genome sequence of a particular species enables chromosome-specific oligo-probes to be created, which, in many cases, constitute invaluable tools that allow the comparative studies of different plant genera [101]. Nowadays, cooperation between molecular and cytogenetic methods is growing closer. The introduction of methods that enable the physical localisation of DNA sequences in the cells with a preserved three-dimensional structure, together with immunocytochemistry, that allows for protein localisation, has had a great impact on gaining a better understanding of the structure and function of cells [259,260]. The use of novel CRISPR imaging methods in plant research [261] has opened new perspectives in the studies of chromatin dynamics *in vivo*.

## Data Availability

Not applicable.
